# Building accountability into the Internet of Things: the IoT Databox model

**DOI:** 10.1007/s40860-018-0054-5

**Published:** 2018-01-27

**Authors:** Andy Crabtree, Tom Lodge, James Colley, Chris Greenhalgh, Kevin Glover, Hamed Haddadi, Yousef Amar, Richard Mortier, Qi Li, John Moore, Liang Wang, Poonam Yadav, Jianxin Zhao, Anthony Brown, Lachlan Urquhart, Derek McAuley

**Affiliations:** 1 School of Computer Science, University of Nottingham, Nottingham, UK; 2 Dyson School of Design Engineering, Imperial College London, London, UK; 3 School of Electronic Engineering and Computer Science, Queen Mary University of London, London, UK; 4 Computer Laboratory, University of Cambridge, Cambridge, UK; 5 Horizon Digital Economy Research Institute, University of Nottingham, Nottingham, UK

**Keywords:** GDPR, Accountability, Internet of Things (IoT), IoT Databox

## Abstract

This paper outlines the IoT Databox model as a means of making the Internet of Things (IoT) accountable to individuals. Accountability is a key to building consumer trust and is mandated by the European Union’s general data protection regulation (GDPR). We focus here on the ‘external’ data subject accountability requirement specified by GDPR and how meeting this requirement turns on surfacing the invisible actions and interactions of connected devices and the social arrangements in which they are embedded. The IoT Databox model is proposed as an in principle means of enabling accountability and providing individuals with the mechanisms needed to build trust into the IoT.

## Introduction

The European Union has introduced new general data protection regulation (GDPR), which comes into effect in May 2018 and is explicitly concerned to handle the threat to privacy occasioned by the emerging digital ecosystem.

“Rapid technological developments and globalisation have brought new challenges for the protection of personal data. The scale of the collection and sharing of personal data has increased significantly ... ... ... the proliferation of actors and the technological complexity of practice makes it difficult for the data subject to know and understand whether, by whom and for what purpose personal data relating to him or her are being collected.” [12]

A key driver of this rapid technological development and technological complexity is the Internet of Things:

“an infrastructure in which billions of sensors embedded in common, everyday devices ... are designed to communicate unobtrusively and exchange data in a seamless way ... clearly raises new and significant personal data protection and privacy challenges” [34]

GDPR, thus, seeks to put in place measures to address these challenges. Key amongst them is the *accountability* requirement.

The accountability principle [31] requires any organization that controls personal data processing, which includes collecting, using, retaining, disclosing and/or disposing of it, by wholly or partly automated means for professional or commercial purposes, and which otherwise provides persons with the means (e.g., cloud-based services) for processing personal data for their own household purposes [12, paragraph 18], be able to *demonstrate *compliance with GDPR. Failure to do so may result in ‘administrative fines’ up to ${\EUR }$20,000,000 or 4% of total annual worldwide turnover, whichever is greater. The consequences of ignoring GDPR are severe, and the IoT is no exception.

It might be argued that, while significant, GDPR only applies in Europe. However, if we consider the ‘territorial scope’ of the regulation, it is clear that any such argument is misplaced.

“This Regulation applies to the processing of personal data ... where the processing relates to ... the offering of goods or services to ... data subjects in the Union ... or the monitoring of their behaviour as far as their behaviour takes place within the Union ... ... regardless of whether the processing takes place in the Union or not.” [12]

The accountability requirement has global relevance then, and this paper seeks to articulate what it amounts to in practical terms for the Internet of Things, and to define a computational model that responds to the requirement and thus builds accountability into the IoT.^1^

Were we to define accountability then we would say that it (a) requires any organization controlling data processing to put policies, procedures and systems in place to demonstrate to*itself* that its processing operations comply with the requirements of data protection regulation. This ‘internal’ focus is emphasized by data protection guidance [e.g., [15]], and may be provided for through such tools as privacy impact assessments (PIAs) [35]. Less pronounced at first glance, though equally as important, is (b) the ‘external’ dimension of accountability, which requires that a data processing entity demonstrate to *others*, particularly regulatory authorities and individual data subjects, that its data processing operations comply with regulation. Internal and external demonstrations are not isomorphic. Thus, accountability cannot be reduced to showing that a PIA has been carried out. More is required, especially with respect to making data processing accountable to the individual.

Below we unpack the external data subject accountability requirement and how it has been translated into practical recommendations for IoT developers by the Article 29 Data Protection Working Party [34], which is set to become the powerful European Data Protection Board under GDPR. These recommendations seek to enable individual control over the flow of personal data through the design of computational mechanisms that enable consent as an ongoing matter, make data processing transparent, and permit fine-grained data flow management, online access and data portability. Satisfying the external data subject accountability requirement requires that we surface and articulate hidden aspects of the IoT ecosystem [38]: not only machine-to-machine or M2M actions and interactions but also, and importantly, the social arrangements connected devices are embedded in [26], for it is not only the data collected by Internet-enabled ‘things’ that must be made accountable but also *what* is done with the data and by *whom*.

We outline the IoT Databox model as a means of surfacing device actions and interactions, and the social or cooperative arrangements they are embedded in, to enable accountability. The IoT Databox is an edge device that is intended to be situated within the home, a key sector for IoT development [20]. It collates data from IoT devices, either directly or via APIs, and makes them available to ‘apps’ that enable data processing and actuation. Data processing takes place on-the-box. Moving computation to the data at the edge of the network, rather than data to centralized processing ‘in the cloud’, has a range of potential benefits which are particularly relevant to the IoT and drive the shift to edge and fog computing [18, 30]. These include low latency (data does not have to be moved to and from remote data centres), resilience (actuation does not need to rely on continuous connectivity), efficiency (centralised data processing costs are significantly reduced), and data minimisation (only the results of processing queries are distributed). Making the IoT accountable may, then, have manifold advantages, which also includes opening up data that is currently distributed across manifold silos to innovation on-the-box.

## The external accountability requirement

The external accountability requirement plays a key role in the processing of ‘personal data’, i.e., any data that relate to an identified or identifiable person, including data generated by connected devices. It requires, by definition, that data processing operations are demonstrably complaint with regulation. This includes, but is not limited, to the following.

**Data minimisation** Article 5 GDPR requires that the processing of personal data is *limited* to what is necessary to meet the purposes for which they are collected and is thus conducted under the auspices of the ‘data minimisation’ principle.

**Lawfulness of processing** Article 6 GDPR specifies that data processing should also be lawful. The processing of personal data is considered lawful to the extent that at least one of the following applies. It is necessary for a controller to process personal data (a) in exercising official authority or performing a task carried out in the public interest, (b) complying with a legal obligation, (c) protecting the vital interests of the data subject, (d) pursuing the legitimate interests of an organisation, or (e) fulfilling a contract. Otherwise, processing is only lawful if the data subject has given *consent* to the processing of his or her personal data for *specific* purposes.

**Fairness of processing** Given the manifold grounds upon which processing may lawfully conducted it may sound on the face of it that just about anything goes, especially given the ‘legitimate interests’ clause. However, Article 5 GDPR also specifies that data processing must be *fair*.

“Fairness generally requires you to be transparent—clear and open with individuals about how their information will be used. Transparency is always important, but especially so in situations where individuals have a choice about whether they wish to enter into a relationship with you.” [16]

Consent, thus, becomes a key ingredient in the processing of personal data, especially where consumer-oriented IoT devices and services are concerned, insofar as it makes data processing transparent and allows individuals to make informed choices.

**Information to be provided to the data subject** The processing of personal also requires certain information be provided to the data subject. This includes the specific purposes of data processing, what data are required, and by whom. Article 13 GDPR also requires that data subjects be informing of any *other* *recipients* of their data and the legitimate interests those recipients pursue, including the *transfer* *of data* to an international organisation or third country for processing (ibid.). If the data are to be transferred then individuals must be informed of the ‘safeguards’ that have been put in place to provide effective legal remedies for data subjects and/or an ‘adequacy decision’ by the EU on the level of protection offered by third country. Individuals must also be informed of any *further processing* of personal data, if those purposes are ‘incompatible’ with those for which they were originally collected [33]. GDPR, thus, renders the international distribution of data processing and data reuse accountable to the data subject.

**Data subject rights** Individuals should, wherever possible, also be informed as to the period for which data will be stored and, in accordance with Article 15, should be able to *access* their data via a secure remote system that enables individuals to *export* their data in a ‘structured commonly used machine-readable format’ as per the right to data portability (Article 20). Other rights that must be made accountable to the data subject include the right to lodge a complaint (Article 15), the right to rectification (Article 16), and the right to be forgotten and to erasure (Article 17). Where automated decision-making, including profiling, is applied then the logic, significance and envisaged consequences of data processing must be made accountable to the individual (Article 13). Furthermore, individuals have the right *not* to be subject to decisions based solely on automated data processing which has significant effects (such as automatic refusal of an online credit application) without the implementation of measures that safeguard their rights, including the right to obtain human intervention and to contest decisions (Article 22).

Consent is not simply a matter of obtaining permission to process personal data then. It requires that data processing be made *accountable* to individuals in terms of specific (legally defensible) purposes that reveal any and all recipients of the data, data transfers (including EU authorisation or legal safeguards), and further processing. The individual’s rights must also be made accountable, including the right to complaint, rectification, and erasure, and the right to online access (wherever possible) and data portability. Automated processing producing legal effects must also be made accountable to individuals and measures put in place that safeguard their rights, including the right to human intervention. These requirements must be articulated in an ‘intelligible and easily accessible form, using clear and plain language’ (Article 12), and ‘at the time when personal data are obtained’ (Article 13).

Satisfying the external accountability requirement is challenging in the IoT, and not only due to the fact that data processing is routinely distributed across an ‘unobtrusive’ and ‘seamless’ infrastructure [34] in which connected devices typically lack user interfaces and the communication of data is invisible. Challenging too is the shifting status of the accountability requirement itself. Something which has traditionally been construed of in engineering terms as a ‘non-functional’ requirement—a matter of providing information to people (e.g., via terms and conditions or privacy notices)—is shifting under GDPR into a ‘functional’ requirement and something that must, therefore, be *built into* the IoT.

The emphasis GDPR puts on the “information to be provided where personal data are collected from the data subject” (Article 13) no doubt bolsters the non-functional view. However, rights to do with online access and data portability clearly signal the shifting status of the accountability requirement, and that it extends beyond the initial moment of consent. While information will have to be provided about a raft of processing issues from purpose to recipients, data transfer and automated processing, the demonstration of compliance with the external data subject accountability requirement can no longer be reduced to the provision of information ‘up front’, any more than it can be reduced to a PIA. Accountability will need to be engineered into the IoT, a point underscored by the Article 29 Working Party (WP29) and the practical recommendations it proposes to manage the ‘data protection risks that lie within the ecosystem of the IoT’ by ‘implement[ing] privacy and data protection *in* products and services’ [34].

## Implementing the external accountability requirement

One of the key risks that attaches to the IoT from a European perspective is the potential for an opaque infrastructure of connected devices to ‘dehumanise’ the world, ‘alienate’ people, and ‘reduce human freedom’ [29]. This is particularly acute in a domestic context, which is seen to constitute a ‘mini IoT environment’ in its own right, capable of revealing its inhabitants’ lifestyles, habits and choices. Ensuring that end-users fully understand ‘the role, functioning and impact IoT services can have on their lives’ thus becomes a critical challenge (ibid.), which the external accountability requirement seeks to address. More than that, however, it seeks to put end-users in control. Accountability is not simply about explaining the IoT to people [9], it is about giving people the tools to *exercise* *control*.

“User empowerment is essential in the context of IoT. Data subjects and users must be able to exercise their rights and thus be ‘in control’ of the data at any time according to the principle of self-determination” [34]

In addition to furnishing end-users or individuals with the information required by GDPR, WP29 recommends that control turn on the implementation of a range of awareness mechanisms. This recognizes that, at the current moment in time at least, communication between devices in the IoT ecosystem often occurs ‘without the individual being aware of it’, which in turn makes it ‘extraordinarily difficult to control the generated flow of data’. The lack of awareness increases the risk of ‘excessive self-exposure’ and ‘functional creep’ as data flows invisibly around the ecosystem. It is further recognized that ‘classical mechanisms’ for promoting awareness are difficult to apply in the IoT, given the seamless character of communications and the current inability for connected devices to make the data they generate ‘reviewable by the data subject prior to publication’. WP29, thus, recommends that a number of practical measures be implemented to increase awareness and reflexively put users in control of the flow of data in the IoT. In addition to implementing adequate security measures, these include:

**Providing granular choice over data capture** Device manufacturers must provide users with granular choices over data capture. The granularity should concern not only the category of collected data, but also the time and frequency at which data are captured. As a feature of granular choice, it is also recommended that devices ‘inform’ users when they are active, e.g., via a physical interface to a device or by broadcasting a signal on a wireless channel, and similar to the do not disturb feature on smartphones, that IoT devices offer a ‘do not collect’ option to quickly disable data collection.

**Limiting data distribution** In keeping with the data minimisation principle and purpose limitation, IoT devices should limit the amount of data leaving devices by transforming raw data into aggregated data and deleting raw data as soon as the data required for processing has been extracted. As a principle, deletion should take place at the nearest point of data collection of raw data and where possible directly on the device.

**Enforcing local control** To ‘enforce user control’, IoT devices should enable local controlling and processing entities allowing users to have a clear and transparent picture of data collected by their devices and facilitating local storage and processing without having to transmit the data to the device manufacturer. Furthermore, IoT devices should provide tools enabling users to locally read, edit and modify the data before they are transferred to any data controller.

It is also recommended, in keeping with GDPR (Article 7), that users should be able to revoke consent and that the tools provided to register this withdrawal should be ‘accessible, visible and efficient.’ Such tools should allow users to continuously withdraw their consent ‘without having to exit the service provided’ by connected devices. Furthermore, and where relevant (e.g., with respect to smart appliances), in withdrawing users should still be able to *use* the device in ‘unconnected’ mode.

The controls recommended by WP29 may sound severe but are not dissimilar to the recommendations of the Federal Trade Commission (FTC), the chief agency tasked with protecting personal data in the US. Accordingly, the FTC proposes a number of practical measures to put the individual in control of personal data generated by IoT devices. These include the implementation of management portals or ‘dashboards’ that enable users to configure IoT devices; ‘privacy menus’ enabling the application of user-defined privacy levels across all of their IoT devices by default; the use of icons on IoT devices to ‘quickly convey’ important settings and attributes, such as when a device is connected to the Internet, and to enable users to quickly ‘toggle the connection on or off’; and the use of ‘out of band communications’ to relay important privacy and security settings to the user via other channels, e.g., via email or SMS.

“Properly implemented, such ‘dashboard’ approaches can allow consumers clear ways to determine what information they agree to share.” [10]

Clearly, there is some resonance between the FTC recommendations and the granular choice measures proposed by WP29, insofar as both are concerned to put computational mechanisms in place that allow end-users to understand data collection and control the flow of personal data in the IoT ecosystem. There is agreement too on the ‘relevance and importance’ of minimising data collection and that greater transparency would ‘help customers and businesses by promoting trust in the burgeoning IoT marketplace’ (ibid.), though we note that there are no overarching principles of data minimisation or transparency in US data protection law [14].

Nonetheless, seen through the lens of key agencies tasked with implementing data protection in Europe and the US, satisfying the external accountability requirement becomes a matter of enabling individual control over the flow of personal data through the design of computational mechanisms that provide for consent as an ongoing matter, make data processing transparent, and permit fine-grained data flow management. In Europe that requirement also extends to computational mechanisms which enable online access and data portability, and more radically that ‘local processing entities’ be implemented to *enforce* control.

One direct implication of the local control recommendation is that a great deal of the IoT data processing that currently takes place in the cloud is moved to the edge of the network.

“The edge of the Internet is a unique place ... located often just one wireless hop away from associated ... devices, it offers ideal placement for low-latency offload infrastructure to support emerging applications ... It can be an optimal site for aggregating, analysing and distilling bandwidth-hungry sensor data ... In the Internet of Things, it offers a natural vantage point for ... access control, privacy, administrative autonomy and responsive analytics.” [3]

Moving data processing to the edge might not only minimize but entirely dispense with the distribution of personal data and the privacy threat that accompanies its distribution. In doing so, there is not only the added benefit of low-latency offload, but resilience in actuation (should the broader network fail), and a significant reduction in data processing costs to processing entities. It may also be the case that in moving to the edge to meet the external accountability requirement, we can open up personal data for innovation in privacy-preserving, trust-building ways.

## Accountability at the edge: the IoT Databox model

Under GDPR the external accountability requirement puts the principle of self-determination into practice and thus requires that *consent* be built into the IoT as an ongoing matter, which means consent can no longer be reduced to ticking a box on a device manufacturer’s or service provider’s remote website; that data processing is *transparent*, and provided for through information clearly articulating specific purposes, recipients, transfers, and the logic, significance and consequences of automated processing; that data collection is *minimal* and involves only that which is needed to meet the purposes of processing; and that individuals be able to *access* their data online and *export* it. Furthermore, it is recommended that external accountability be implemented through computational mechanisms that allow individuals to exercise *granular choice* over data collection; *limit data distribution* and keep raw data as close to source as possible; and permit *local control* allowing individuals to review the results of processing operations prior to ‘publication’ or distribution. Limiting data distribution and permitting local control inevitably nudges solutions enabling external accountability to the edge of the network.

**Fig. 1:**
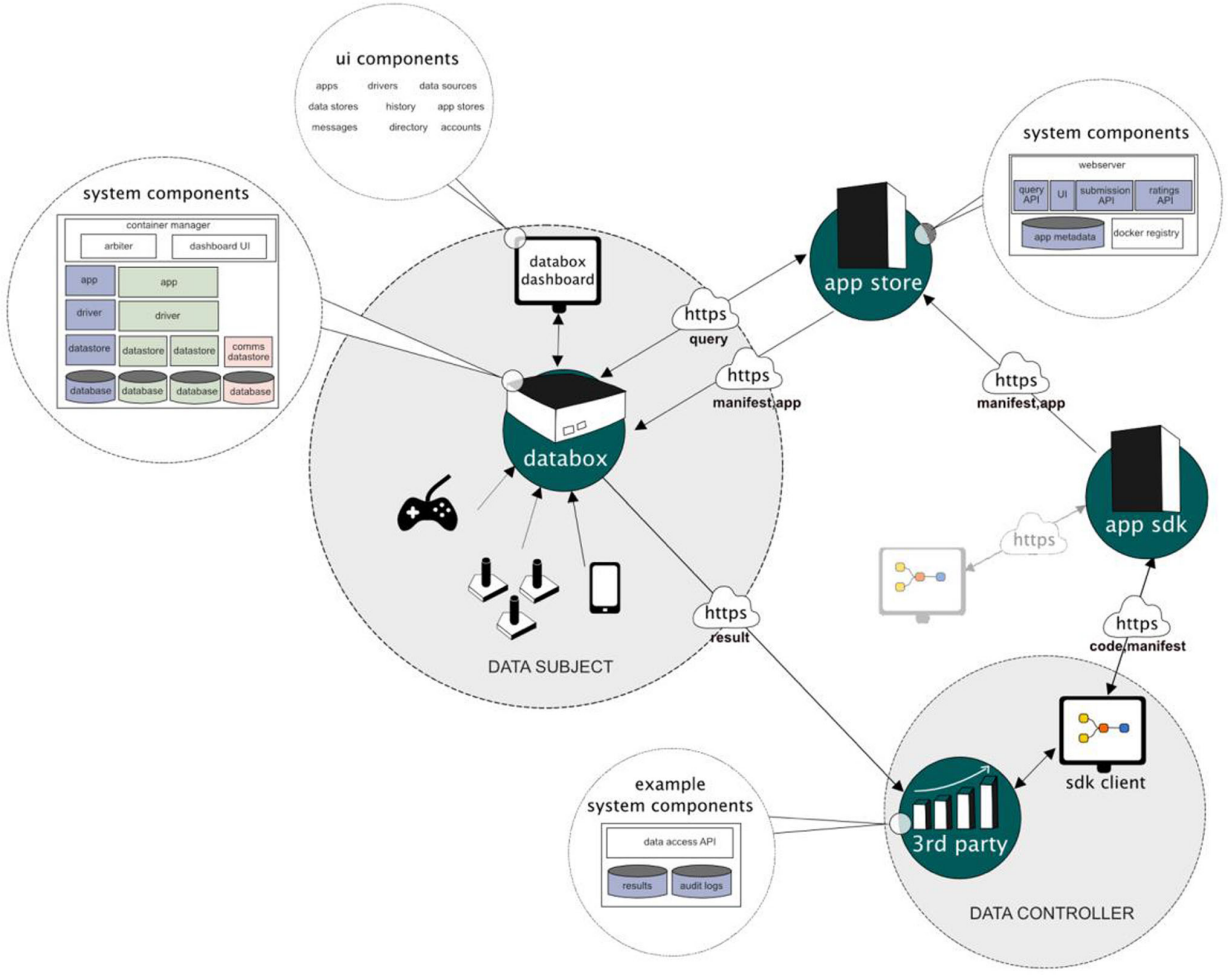
Enabling external accountability: the IoT Databox Model

### Origin and evolution of the model

The IoT Databox model provides an in principle means of implementing the external accountability requirement. The model extends the Databox concept [2] to incorporate the IoT. The Databox concept posits a physical device as a gateway to a distributed platform and is predicated on the ‘Dataware model’, which sought to develop a business to consumer (B2C) service-oriented architecture providing a new wave of personal digital services and applications to individuals [19]. This model posits a ‘user’ (by or about whom data is created), ‘data sources’ (e.g., connected devices, which generate data about the user), a ‘personal container’ (which collates the data produced by data sources and can be accessed via APIs), a ‘catalogue’ (which allows the user to manage access to the personal container), and ‘data processors’ (external machines exploited by parties, or ‘data controllers’ in GDPR terminology, who wish to make use of the user’s data in some way).

The Dataware model is a logical entity formed as a distributed computing system. Data processing involves requests being sent to the catalogue, which are approved or rejected by the user. If approved, the catalogue issues a processing token to the data processor for permitted requests. The processor presents the token to the personal container, which accepts the token, runs the processing request on the relevant data sources, and then returns processed results to the data controller. The Dataware model represents a distinctive approach to personal data processing, that not only seeks to enable user control but also data minimization. Thus, the Dataware model takes a significant step towards implementing the local control recommendation, minimising data sharing to the *results* of processing. The raw data remains ‘on the box’ under the users control.

The Dataware model is currently being reconfigured around the Databox concept, which embeds the Dataware model in a physical object situated in the physical environment (e.g., a networked mini-computer in the home) under the direct control of the individual. It allows the individual to collate data from an array of data sources in a single place and allows the individual to control access to them. Data from individual data sources is stored in ‘data stores’, i.e., containerised, application-specific, processes [e.g., 22] that reduce the attack surface and management problems associated with general purpose operating systems.

**Fig. 2:**
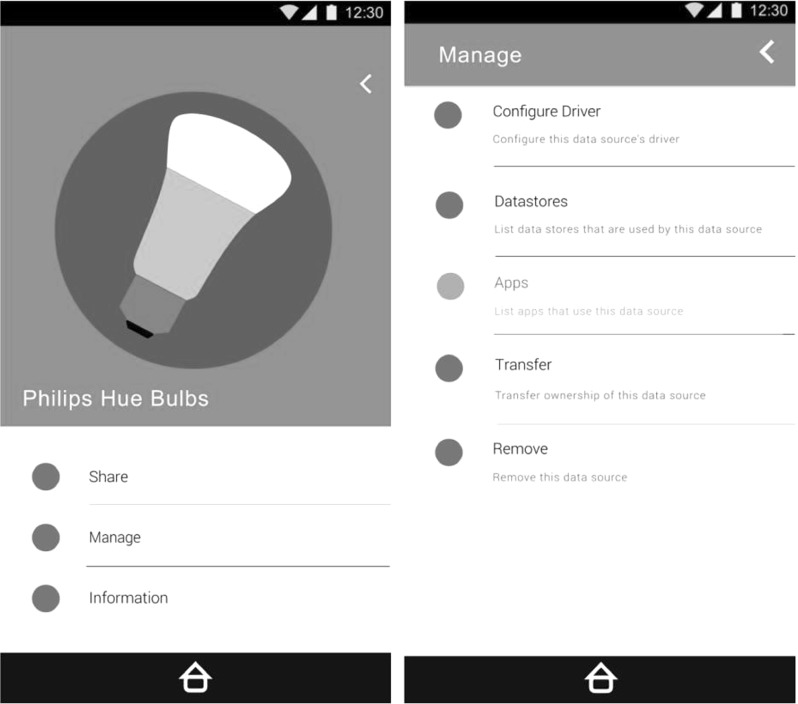
The databox dashboard

### Architecture of the model

Architecturally the IoT Databox model consists of three key components: the *Databox*, an *app store* (of which there may be many), and third party *processors* (Fig. 1). Data processing is done through apps that run on the Databox and are publically distributed by developers via the app store. The Databox itself is a small form factor ($\times$86 or ARM) computer consisting of a collection of containerised system services including the *dashboard* (Fig. 2), which provides Databox users with a range of management functions including:

- Creating *User Accounts* on the Databox and activating sharing permissions (e.g., that consent from all users of shared resources is required for delete actions).
- Adding *Data Sources* to the box; including assigning ownership to data sources, annotating data sources (e.g., smart plug X is ‘the kettle’), and sharing data sources with other Databox users.
- Configuring *Drivers* to enable data sources to write to data stores.
- Managing *Data Stores*; including sharing stores with other Databox users, and redacting, clearing, or deleting stores.
- Accessing *App Stores*; apps are recommended by the box based on available data sources but individuals can also search for, download, and rate apps.
- Sharing *Apps*, with other users within the home and between distributed Databoxes in other homes; the Dashboard also allows apps to be updated and deleted.
- Receiving *Notifications*; including the results of data processing prior to distribution, sharing requests, app updates, resource contention, etc.
- *Auditing* data processing operations; including all accesses to data stores, and any data transactions.

The app store is a cloud-based service, interacted with using standard internet protocols (principally HTTPS). It consists of a web server that provides the app store UI supporting human interaction, and a query API providing for programmatic (machine-based) interaction. The app store manages a docker repository [8] of apps, which are uploaded via the app submission API and indexed by associated metadata.

### App development

App developers are free to create their own containerised apps as they wish, but the app store provides a dedicated app SDK supporting the app building and publication process. This is a cloud-hosted visual code editor based on IBM’s open source Node-RED [24], which utilises a flow-based programming paradigm in which black-box processes called ‘nodes’ are connected together to form applications called ‘flows’.

**Fig. 3:**
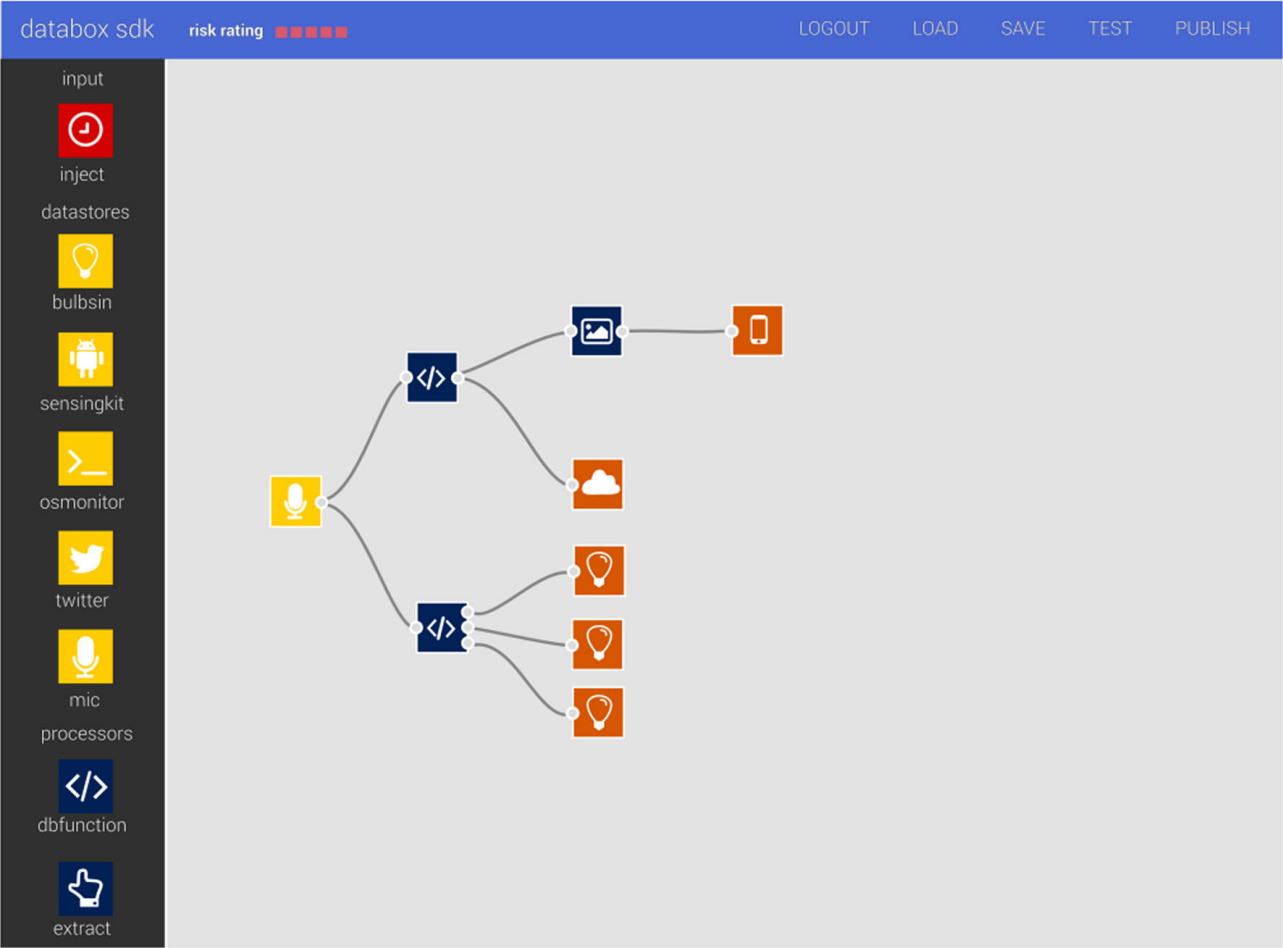
The IoT Databox App SDK

There are three principle node types:*data sources*,*processes* and *outputs*. *Process* nodes are functions that operate on data; they typically have a single input connection and one or more output connections. *Output* nodes typically perform an action, such as actuation, visualisation, or data export. Figure 3 depicts a flow taking the output from a microphone, performs some processing on the data and updates a visualisation, turns on one or more bulbs, and exports the processed data to the cloud. It is composed of a single data source (yellow node), three processes (blue nodes) and five outputs (orange nodes).

The app editor smooths and simplifies the build–test–deploy development workflow; it presents a high-level abstraction (e.g., an app developer can build an app without needing to be familiar with the interoperation between sources, stores and drivers); it provides ‘scaffolding’ to help build an app (e.g., developers can quickly inspect the structure and type of data entering and exiting a node); it provides a full testing environment, where flows are deployed (as containers) and connected to test data; it handles the app publication process by presenting tools for building a ‘manifest’ (Fig. 6) enabling end-user consent and granular choice; and, upon submission, it containerises an app and uploads it to the app store. The SDK also takes care of source code management as all stages of the app development cycle are recorded in a developer’s GitHub account.

**Fig. 4:**
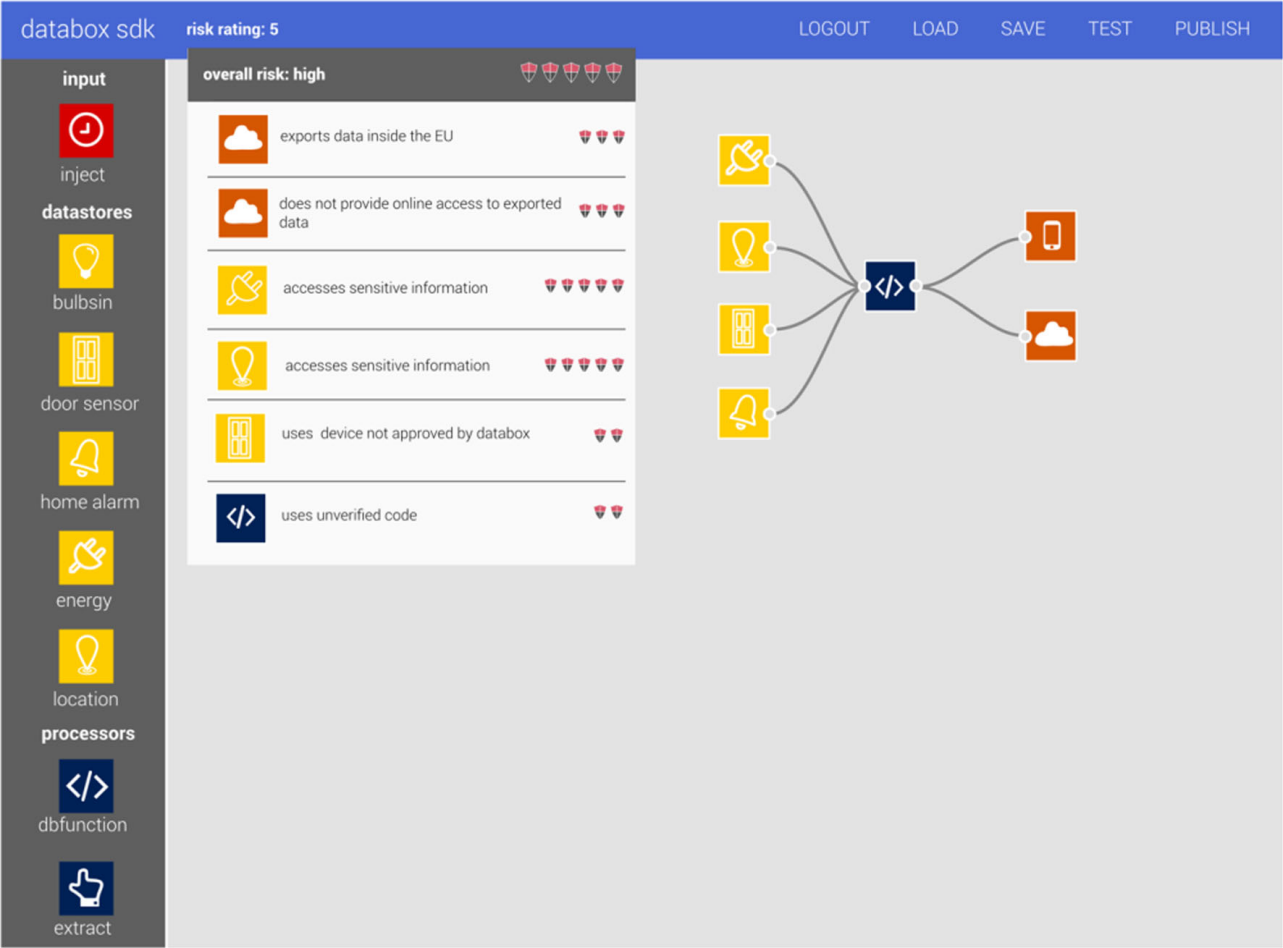
SDK risk rating apps during development

**Fig. 5:**
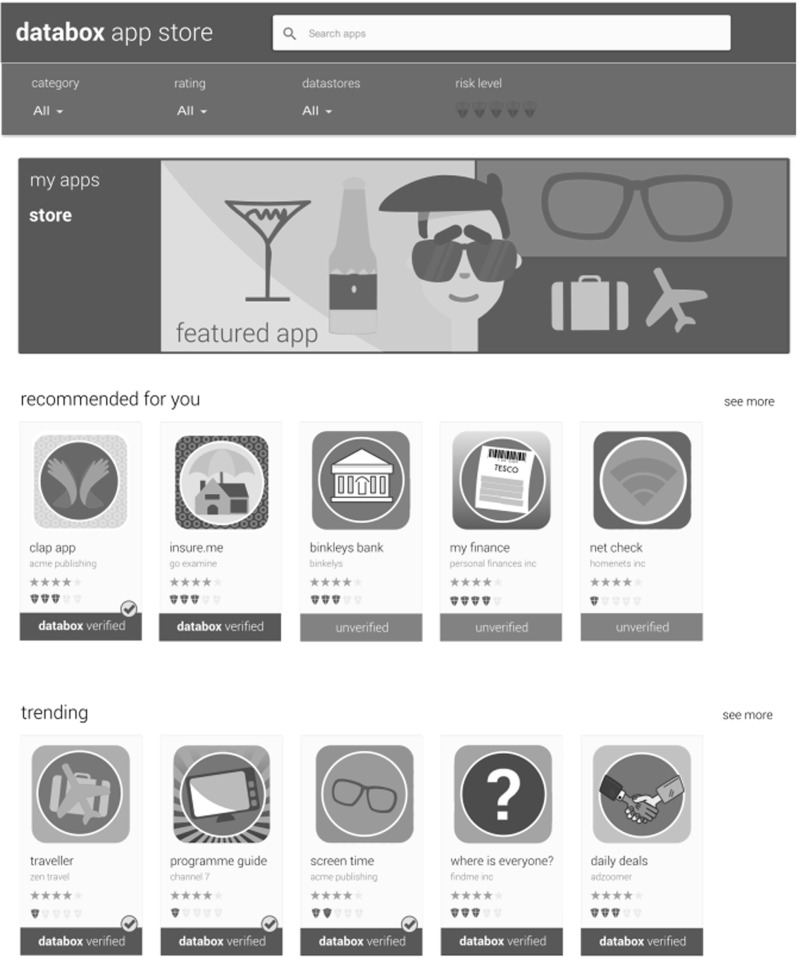
At-a-glance risk (bars) and user ratings (stars)

### Managing risk

Importantly the SDK also seeks to sensitize app developers to the potential risks that accompany personal data processing. We differentiate between three types of risk: *legal risks* associated with GPDPR, particularly those implicated in taking data off-the-box including data export within the EU, outside the EU, transfer to other recipients, the provision of adequacy decisions or safeguards, and access; *technological risks*, including apps that use devices that have not been validated by the SDK, use unverified code, or physically actuate essential infrastructure or potential dangerous devices in the home; and *social risks*, including apps that access sensitive information or produce results that may be deemed sensitive (as articulated, for example, by the notion of ‘special categories’ of personal data in Article 9 GDPR).We take the view that app developers should be clear about the nature and level of risk of posed by an app and provide precise information about the risks they potentially expose users to.

We appreciate that identifying risk is challenging, given that it can be introduced by any individual component of a system (both hardware, such as sensors/actuators, and software, such as drivers and apps) as well as arbitrary combinations of the two in particular operating contexts. Though by no means infallible, the SDK generates a risk rating for apps, based on the aggregate risk of the nodes from which it is composed. Each node in the development environment has a pre-defined spectrum of risk attached to it. The final risk rating assigned to the node will sit within this spectrum, and will be determined by how nodes are configured (e.g., the hardware they work with, the proposed data rate, the particular actuation to be performed, etc.). The SDK provides developers with a view on potential risk *in the course of* app construction (Fig. 4) in a bid to reduce risk in the IoT ecosystem. The risk rating of apps is also made available to users on the app store (Fig. 5) to further motivate and drive the development of low risk and even no risk apps that do not export data, provide users with granular choice over data sampling and reporting frequency, provide online access if apps take data of the box, clearly flag that they actuate essential infrastructure in the home (e.g., central heating or windows and doors), and exploit accredited hardware and trustworthy software. Low-risk apps are visibly ‘checked’ in the app store to display their Databox accredited status.

The risk rating assigned by the SDK is reflected in the app store once uploaded. For apps built outside the SDK, the app store reviews and rates them based on features and information provided, e.g., the absence of an API providing users with access to their data would result in a high-risk rating if data were taken off-the-box by an app. Apps may also be posted on the app store with an ‘unverified’ status, in which case their risk rating will also be high. However, an app cannot be posted on the app store or installed on the IoT Databox without a ‘manifest’ being in place, and data (i.e., the results of processing) cannot be transferred to a controller’s processors without a manifest being completed by the individual or data subject.

### Enabling consent and granular choice

**Fig. 6:**
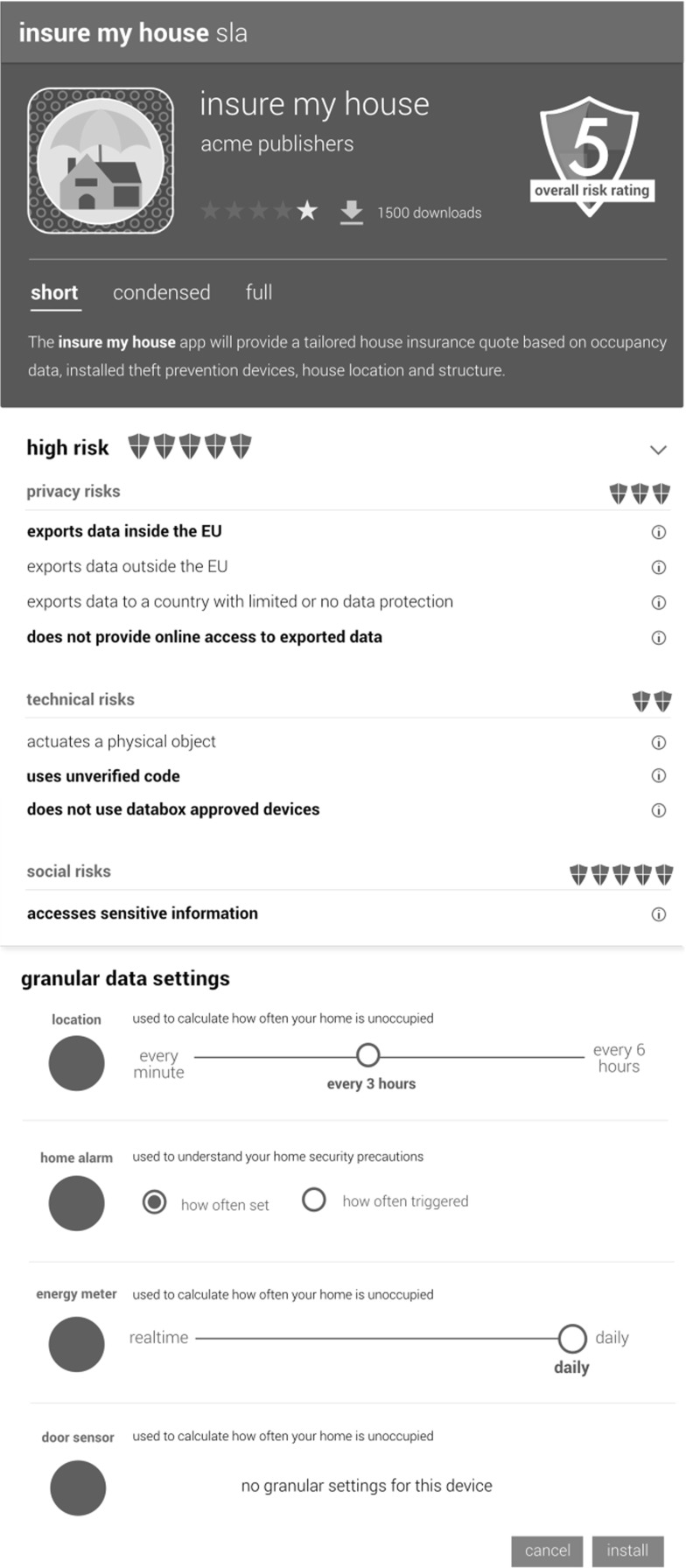
Manifest enabling consent and granular choice

Manifests are ‘multi-layered notices’ [32], which (a) provide a *short* description of the specific purpose of data processing, (b) a *condensed* description providing the information required by GDPR, and (c) *full* legal terms and conditions. The IoT Databox also adds app information to the short description, including user ratings and an app’s risk profile, and enables control to be exercised over data collection at device level (Fig. 6). Multi-layered notices are, thus, transformed into dynamic, user-configurable consent mechanisms that surface and articulate who wants to access which connected devices and what they want to process personal data for. Thus manifests make specific socio-technical data processing arrangements, implicating connected devices, data controller’s and their processors accountable to individuals and available to local control.

**Fig. 7:**
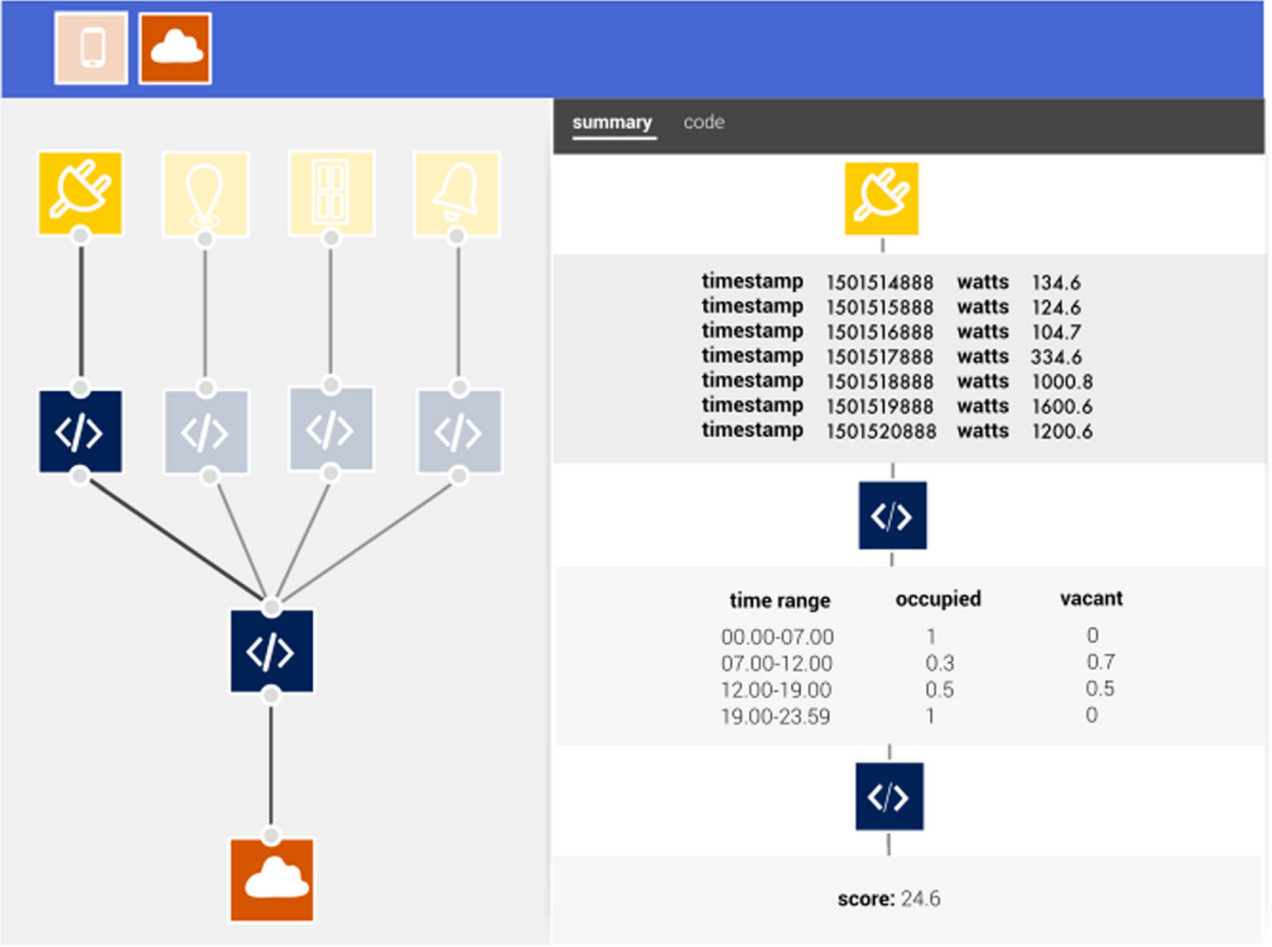
Building runtime accountability into apps

Manifests provide an easy to read description in clear and plain language of the data sources an app will use, and the risks that attach to using the app. They also allow users to exercise fine-grained granular choice over data collection, selecting just which data sources may be used and, insofar as connected devices permit, at which sampling frequencies data will be gathered. Once a manifest has been configured by the individual and has been installed it assumes the status of a service level agreement (SLA), which the IoT Databox transforms into a set of machine readable policies that enforce a data processor’s access to the particular data sources agreed upon by the individual and regulates subsequent data processing operations. Apps, like data stores, run within isolated containers and interact with data stores to perform a specified (purposeful) task defined in the SLA. Thus apps may query data stores, write to a communications data store that sends query results to external machines, or write to a connected device’s store to perform actuation. Data stores record all actions performed on them (queries, external transactions and actuation) in an audit log. Access to data stores are enforced by the ‘arbiter’, which issues and manages the use of access tokens.

### Making data processing accountable

As more and more connected devices find their way into the home, and an increasing array of apps consume personal data and operate on users’ behalf, then we expect the ability to inspect *what* has happened and *why* will be a necessary feature of app usage. For example, I know my health insurance app provides quotations based on my activity, grocery shopping, location, and financial data, but just how has it arrived at the quotes that it does? Alternatively, one might wonder why the radiators in the living room were set to the maximum at 3 A.M. yesterday, or why a large order of toilet roll has appeared on the doorstep? Whatever the particular case, GDPR makes it clear that the logic, significance and consequences of automated processing be made accountable to individuals. This may in part be provided in the information contained in consent mechanisms as a preface to app use but, as the above examples indicate, there is also a need to build *runtime* *accountability* into the IoT.

To enable runtime accountability, and in addition to dashboard notifications, apps created in our SDK are bundled with an inspection interface that surfaces how an app ‘operates’, i.e., how data flows through an app and how some action or decision is arrived at, in order to support real-time interrogation by users. By way of example, Fig. 7 illustrates how data is processed as it moves along the flow path. The path summarises how energy data is used as part of a calculation of a final score sent back to a third party to generate a home insurance quote. The timestamp and watts listing displays the raw data from the energy data source. When it is subsequently processed by the first function node (in blue) it is transformed into an occupancy matrix for times of the day, with the values for house ‘occupied’ and ‘vacant’ represent probabilities in the range from 0 to 1. Finally these data, alongside data from other data sources implicated in the other flow paths (location, alarm and door sensor data), are provided to the final function to produce an overall score. As with our attempt to convey potential risk, this is a nascent first step towards enabling runtime accountability. Nonetheless we think it an important area of research and topic of future work, particularly with respect to how an app’s operations are conveyed to users, given the emphasis placed on automated processing by GDPR.^2^

## Responding to the privacy challenge

GDPR requires that external data subject accountability be built in to the digital ecosystem in a bid to respond to the privacy challenges occasioned by the emerging digital ecosystem. The Article 29 Working Party provides a number of practical recommendations as to how data protection can be implemented in the IoT in particular. Together, these legal requirements and recommendations suggest that meeting the external data subject accountability requirement is a matter of enabling individual control over the flow of personal data through the design of computational mechanisms that (a) provide for consent as an ongoing matter, (b) make data processing transparent, (c) permit fine-grained data flow management, (d) allow online access and data portability, and (e) exploit local processing entities to enforce control. The IoT Databox model provides an in principle means of meeting the external accountability requirement insofar as it provides tangible computational mechanisms that address these concerns.

**Consent** The requirement here is not only that users be able to consent to data processing in the IoT, but also they can do so as an ongoing matter and thus revoke consent. Consent is provided for by the IoT Databox through dynamic multi-layered notices, which do not sit at some remove from data processing (e.g., on a remote website) but are installed on-the-box where processing occurs. That means they can also be uninstalled at any time by the user and data processing be terminated at will. We cannot guarantee that a connected device will still work, as per WP29 recommendations, but that is a matter for device manufacturers to address.

**Transparency** The information required by GDPR to make data processing transparent—including purpose specification, recipients, transfers and salient details of automated processing—is also provided by multi-layered notices. Additionally, the IoT Databox provides a raft of transparency mechanisms articulating the potential risks that attach to apps, dashboard notifications allowing users to review the result of data processing, runtime accountability mechanisms tracing data processing operations, and audit mechanisms that allow users to inspect the historical operations of apps on the box.

**Fine-grained data flow management** Multi-layered notices also enable users to exercise granular choice over data collection, insofar as connected devices provide a range of data sampling frequencies. It is also the case that well-designed apps can support granular choice in offering users a range of reporting frequencies (e.g., continuous, hourly, daily, weekly, monthly) built into multi-layered notices. The IoT Databox additionally supports fine-grained data flow management in limiting and minimising data distribution, aggregating data on the box and only returning the results of processing to a controller. Raw data thus remains on-the-box subject to user control.

**Access and portability** Insofar as raw data remains on-the-box, and audit mechanisms log all processing operations, then data portability is non-issue in the IoT Databox model: the data are always available and the results of specific queries can always be recovered. Providing access to data that has been transferred off-the-box is more problematic. Minimally data controllers will have to provide a secure data endpoint and an encrypted connection if they wish to take any data off-the-box, which the box will monitor. While access is a legal requirement under GDPR, we cannot enforce it. We can encourage it, however, by attaching relatively high risk profiles to apps that take data off-the-box but do not provide online access and, where possible, recommending alternatives.

**Local control** Situated at the edge of the network, the IoT Databox enables local control, which is seen as key to user empowerment. Taking computing to the data, rather than data to the computing, provides individuals with strong privacy management mechanisms. It also has potential computational advantages, decreasing latency, enhancing resilience insofar as devices only need to talk to a local box rather than a remote server, and decreasing network traffic insofar as this approach is adopted at scale, not to mention greater availability and access to data [13].

In limiting and even eradicating the need for remote data processing, edge solutions may foster broad societal trust and innovation, giving users the confidence to allow applications to access personal and even private data with the assurance that it will not be distributed but stay on the box or otherwise be limited the results of a query, which may itself be terminated. The possibility turns, of course, on it being possible to route IoT devices through such devices as the IoT Databox. That is, on device manufacturer’s enabling local control on their products. However, even if they do not, and instead provide users with APIs for apps to access their data, the data itself can still be collated on the IoT Databox and opened up to broader use.

### Fit with the state of the art

We are not the first to espouse the virtues of privacy-preserving platforms. A raft of Personal Data Stores (PDS) have emerged over recent years. Many provide users, like Mydex [23], with encrypted data stores distributed across the cloud against which a wide a variety of third party applications can be run. Despite the phenomenal growth in PDS solutions—the WEF reports that more than one a week was launched between January 2013 and January 2014 alone [37]—widespread public uptake has been problematic. Ironically, a recent report suggests that this is due to ‘perceptions of privacy and security risks’ individuals attach to storing their personal data *in the cloud* [17].

Alternatives are provided by solutions such as openPDS and HAT. OpenPDS [6] is hosted on either a smartphone or an internet-connected hard drive situated in the home. OpenPDS provides users with a centralized location for storing personal data and exploits the ‘SafeAnswers’ approach [7] to compute third-party queries inside a software sandbox within the user’s PDS returning, like the IoT Databox, only the results of processing not the raw data. HAT [36] provides users with a personal container that also stores data client-side. Purpose-built ‘data plugs’ fish personal data from APIs and deposit it into a user’s personal HAT container. HAT-enabled applications access data through ‘data debits’, which permit access to raw data in return for specific services. The primary purpose of HAT is to create a marketplace that redresses the current asymmetry in data harvesting and builds users into the personal data value chain.

The MyData initiative [25] takes a different approach again. It does not provide a PDS solution, but instead seeks to enable consent management. MyData thus provides a digital service that focuses on managing and visualising data use authorisations, rather than storing data itself. It seeks to encourage service providers to build MyData APIs, which enable their services to be connected with MyData accounts. MyData APIs enable interaction between distributed data sources and data users, and the MyData account provides users with a single hub for granting services the authority to access and use their personal data. While the MyData account lets individuals activate or deactivate the sharing of specific data flows and lists currently active authorisations, it does not put further measures in place to limit access and minimise data distribution.

Both MyData and HAT expose raw data to applications and thus fail to limit the potential ‘function creep’ [34] that currently characterises data processing in the IoT and results in personal data flowing unfettered around the ecosystem. Both openPDS and the IoT Databox put severe constraints on the flow of data, minimising it to the results of data processing. While this too has the potential to expose users in ways they might not wish, e.g., through running multiple applications from a developer that allows them to build rich profiles from an array of returned results, the risk can be mitigated, e.g., through applications that monitor app usage and notify users as to the potential inferences that can be drawn from combined processing results.

Although openPDS is ‘aligned with the European Commission’s reform of the data protection rules’ [6], the IoT Databox seeks to respond directly to the external accountability requirement mandated by GDPR. In doing so, it provides users *and* developers with a more extensive set of tools for GDPR compliant data processing in the IoT. Along with a suite of computational mechanisms enabling consent, fine-grained data flow management and transparency, not only of what data is required for what purpose by whom but also of runtime operations and processing results prior to distribution, the IoT Databox provides a dedicated application development environment fostering a culture of accountability in the IoT.

Furthermore, the IoT Databox moves beyond the ‘individual-centric’ [7] approach adopted by openPDS and other solutions. As [4] point out, most personal data do not belong to a single individual but are *social* in nature, especially in the IoT where connected devices are embedded in the fabric and furniture of buildings. The ability to share devices, data, and applications within and between homes, and to collectively as well as individually manage data processing, is also a unique feature of the IoT Databox model.

## Conclusion

The European Union has introduced new data protection regulation (GDPR) that comes into force in 2018. The regulation is largely motivated by the effects of digital technology, which make it difficult for individuals or data subjects to know and understand whether, by whom and for what purpose personal data are being collected and processed. The European data protection agency WP29 views the IoT—an infrastructure designed to communicate and exchange data unobtrusively and in a seamless way—as particularly problematic, raising new and significant privacy challenges. The regulation has global reach and applies regardless of whether or not data processing takes place in the Union if it leverages personal data to monitor or deliver goods and service to individuals in the Union. It is also punitive, exacting heavy fines on data controller’s or parties who process personal data, and otherwise provide individuals with the means to process personal data for household purposes, who flaunt the regulation.

A key pillar of the regulation is the accountability requirement:

“The controller shall be responsible for, and be able to demonstrate compliance with ‘accountability’.” [12]

Accountability has two distinct aspects to it. One ‘internal’, requiring that data processing entities demonstrate to themselves that their operations comply with the regulation. The other ‘external’, requiring that data processing entities demonstrate to others, particularly supervisory authorities and data subjects, that their operations comply with the regulation. The demonstrations are not equivalent, and cannot be provided for in the same ways. The external data subject accountability requirement in particular requires a raft of measures be put in place to enable consent, make data processing transparent, permit fine-grained data flow management, online access and data portability. Recommendations from WP29 for IoT developers also advocate providing granular choice, limiting data distribution, and enabling local control to enforce user control over data processing.

These mandated measures and recommendations mark the shifting status of the external data subject accountability requirement, from non-functional and the provision of information to functional and the implementation of computational mechanisms that *build accountability into the IoT*. This paper has sought to address how this might be achieved. We have sketched out the external data subject requirement as laid down by new regulation, and salient recommendations provided by WP29 for making the IoT GDPR compliant, and how these might be built into the ecosystem via the IoT Databox model.

The model builds on prior work on B2C service oriented architectures to enable a new wave of personal digital services and applications to individuals. The IoT Databox is an edge solution that implements the local control recommendation and collates personal data on a networked device situated in the home. It meets the external accountability requirement by surfacing the interactions between connected devices and data processors [9], and articulating the social actors and activities in which machine-to-machine interactions are embedded through a distinctive range of computational mechanisms [26]:

**Databox** A physical networked device situated in the home enabling users to exercise direct control over IoT devices and to manage both internal (within the home) and external (third party) access to the data they generate. The IoT Databox puts the principle of data minimisation into effect, taking computing to the data, and limiting the potential for excessive self-exposure and function creep in executing processing locally and only returning the results of third party queries.

**App store** A familiar environment enabling users to access data processing services and providing resources to make informed choices about the services they wish to use, including app verification, risk ratings, and feedback from the user community. The app store puts the principle of self-determination into effect, and allows individuals to exercise direct control over the specific data processing operations that run on the Databox.

**Apps** Apps provide a key interface for articulating the transparency requirements of GDPR in terms of manifests, which articulate who wants what data for what purposes along with recipients of the data, data transfers, and the nature of any automated processing that may be applied. App manifests put the principle of informed consent into effect, and in being dynamic objects (not just text) further allow users to exercise granular choice over data collection to enable fine-grained data flow management.

**Dashboard** The Databox dashboard enables individual and collective management of data processing operations. It allows users to exercise fine-grained control over device, data and app use between Databox users both within and between homes. It enables consent to be exercised in an ongoing manner, including revoking it at any time. And provides further transparency mechanisms on data processing operations, both at runtime and on completion, allowing individuals to terminate third party queries should they wish. The dashboard thus enables individuals to exercise further fine-grained control over data processing and the flow of data.

**SDK** The SDK provides developers with an environment enabling accountability to be built into IoT Databox apps, supporting manifest construction to meet the information requirements of GDPR, enhanced granular choice over data collection, and providing for runtime accountability in surfacing how data flows through an app and how some action or decision is arrived at. The SDK also exploits a risk-based framework to motivate development of GDPR compliant apps providing access to data taken off-the-box.

In adopting the local control recommendation and moving data processing to the edge of the network to ensure the individual can control the flow of personal data, the IoT Databox model may enhance the efficiency of data processing, make actuation more resilient, minimize the impact of IoT traffic on the network, and negate the need for costly privacy regimes. Insofar as it is possible for data processing and data to demonstrably *stay on-the-box* then the IoT Databox model also holds the promise of opening up personal data, giving individuals the confidence to allow data processing across manifold sources of personal data rather than single connected devices.

Nonetheless, we are aware that the IoT Databox model is largely symbolic at this moment in time, a signal of what might be possible if the challenges occasioned by edge [30] and fog [18] computing can be overcome. It is also imperative, as raised in discussion of this paper, that we validate the IoT Databox model. This is a non-trivial task which cannot simply be bolted onto a paper, but will involve manifold evaluations across two key areas covering (a) the system and (b) its use.

We, thus, envisage validating the performance of the IoT Databox model on different hardware, including relatively powerful devices (such as Intel NUCs) and relatively cheap devices (such as Raspberry Pi 3s), using various macro and micro benchmarks. The former includes end-to-end benchmarks which will assess temporal performance of the IoT Databox model on different hardware platforms. The latter will include evaluations of the memory footprint of components as the number of apps, drivers and data sources scale up; read/write performance of data stores as the number of stores scales up; latency and throughput limitations introduced by the networking component; the impact of logging; and any constraints introduced by token minting and validation).

Technical measures are necessary but not sufficient to validate the IoT Databox, it is also imperative that it meets human need. Two stakeholder groups are of particular relevance: industry and end users. We thus envisage verifying IoT Databox utility from industry and end-user perspectives. This will include documenting industry engagement, uptake and use, which is already in progress through the development of project partner use cases. It will also involve deploying the IoT Databox in end-users’ homes and evaluating its use from the mundane perspective of everyday life.

Despite its symbolic status, the IoT Databox model is not a theoretical model. It exists [1, 21], albeit in nascent form and its source code is freely available for widespread use [5]. It enables data controllers and app developers working on their behalf to demonstrate compliance with the external data subject accountability requirement. Its ability to support local computation minimises and even circumvents the widespread threat to privacy occasioned by the IoT. And in circumventing the privacy threat, it opens up new possibilities for exploiting personal data in ways that build consumer confidence, and with it widespread trust, into the IoT.

“Data protection must move from ‘theory to practice’ ... accountability based mechanisms have been suggested as a way [to] ... implement practical tools for effective data protection [31].”

## Footnotes

1 The global relevance of the accountability requirement is further underscored by US legislation. While new data protection law appears to be terminally ‘bogged down’ in the US [27], the accountability requirement is enshrined in existing Fair Information Practices [11], and the Federal Trade Commission has been proactive in championing data subject accountability in the IoT [10]. Add to this the Japanese effort to ‘harmonise’ its data protection regulation with international law [28] and it becomes clear that the accountability requirement is poised to exert considerable force on the IoT, whether developers like it or not.

2 In discussion of this paper, it was asked if the Databox has mechanisms in place to check consistency between the manifest and the actual operation of the app? In the first instance, an app can only access stores to which it has been granted access and this is enforced by the arbiter component. In the second instance, the network component prevents an app from exporting data directly—all data exports are done via the export component and can only occur if permitted by an app’s manifest. In third instance, the audit component logs all data stores accessed by an app and all data exported by app. In the fourth instance, the data subject can inspect the operations of an app (a) by previewing data processing prior to data export, (b) by drilling down into data processing operations in detail, and (c) by inspecting the audit log. In the fifth instance, it may also be possible to build apps that monitor the operations of apps automatically. It was also suggested in discussion that in enabling runtime accountability a potential conflict is revealed between what a third party might want to disclose about the inner workings of data processing (e.g., how an algorithm operates) and satisfying the external data subject accountability requirement. We agree, but GDPR is clear: the logic, significance and consequences of automated processing, including profiling, *must* be clearly accounted for (Article 13) insofar as data processing applies to EU citizens. It would appear that there is no way of resolving potential conflict here other than to comply with the regulation, as a failure to comply with the basic conditions of processing, including *informed* consent, could result in devastating fines of up to ${\EUR }$20,000,000 or 4% of total annual worldwide turnover, whichever is *higher* (Article 83).
